# Supplementary material for: Hybrid Models and Biological Model Reduction with PyDSTool
Source: PLoS Comput Biol. 2012 Aug 9;8(8):e1002628. doi: 10.1371/journal.pcbi.1002628 (PMC3415397; doi:10.1371/journal.pcbi.1002628)
Supplement: Text S4 — Complete source code for the PyDSTool package (version 0.88.120504). Includes API documentation and help files linking to web pages. This file is identical to the current public release on Sourceforge.net. (ZIP) [file pcbi.1002628.s004.zip › PyDSTool/html/PyDSTool.Toolbox.ModelEst.ModelEst-class.html]

xml version="1.0" encoding="ascii"?


PyDSTool.Toolbox.ModelEst.ModelEst


| Home | Trees | Indices | Help | PyDSTool |
| --- | --- | --- | --- | --- |

Package PyDSTool ::
Package Toolbox ::
Module ModelEst ::
Class ModelEst

[hide private]

[frames] | no frames]

# Class ModelEst

source code

```
    object --+    
             |    
common.Utility --+
                 |
                ModelEst
```

---

General-purpose model estimation class.


|  |  |
| --- | --- |
| Instance Methods | [hide private] |

|  |  |
| --- | --- |
| \_\_init\_\_(self, \*\*kw)  x.\_\_init\_\_(...) initializes x; see x.\_\_class\_\_.\_\_doc\_\_ for signature | source code |

|  |  |
| --- | --- |
| fit(self, name, free\_pars, parest=None)  Fit an individual candidate model in its context, peforming parameter optimization on the given free parameters. | source code |

**Inherited from `object`**:
`__delattr__`,
`__getattribute__`,
`__hash__`,
`__new__`,
`__reduce__`,
`__reduce_ex__`,
`__repr__`,
`__setattr__`,
`__str__`


|  |  |
| --- | --- |
| Class Variables | [hide private] |

\_needKeys = `['manager', 'context']`

\_optionalKeys = `['libraries', 'verboselevel']`


|  |  |
| --- | --- |
| Properties | [hide private] |

**Inherited from `object`**:
`__class__`


|  |  |
| --- | --- |
| Method Details | [hide private] |

|  |  |
| --- | --- |
| \_\_init\_\_(self, \*\*kw)  *(Constructor)* | source code |

x.\_\_init\_\_(...) initializes x; see x.\_\_class\_\_.\_\_doc\_\_ for
signature

Overrides: object.\_\_init\_\_: *(inherited documentation)*

  


| Home | Trees | Indices | Help | PyDSTool |
| --- | --- | --- | --- | --- |

|  |  |
| --- | --- |
| Generated by Epydoc 3.0.1 on Fri May 4 15:24:08 2012 | http://epydoc.sourceforge.net |
